# Synchronous cardiac and hepatic echinococcosis in a pediatric patient: a case report

**DOI:** 10.1097/RC9.0000000000000612

**Published:** 2026-06-24

**Authors:** Alwaleed Al-Dairy, Ahmad Al-Bitar

**Affiliations:** a Cardiac Surgery at the Faculty of Medicine, Damascus University, Damascus, Syrian Arab Republic; b Faculty of Medicine, Damascus University, Damascus, Syrian Arab Republic

**Keywords:** cardiac hydatid cyst, cardiopulmonary bypass, echinococcosis, hepatic hydatid cyst, hydatidosis, pediatric, surgical excision

## Abstract

**Introduction:**

Hydatidosis caused *by Echinococcus granulosus* rarely involves the heart (0.5–2%), posing risks of rupture, tamponade, or anaphylaxis. Synchronous cardiac and hepatic disease is exceptionally uncommon in children.

**Case presentation:**

An 8-year-old Arab male presented with abdominal pain, vomiting, fever, and dry cough; there was no prior instability or arrhythmia. Imaging revealed multiple calcified hepatic cysts and a subepicardial cyst compressing the left ventricle (with no coronary involvement). After administration of albendazole (15 mg/kg/day for 2 weeks preoperatively), a staged approach was employed. During cardiac cyst exposure, hypotension developed, necessitating cardiopulmonary bypass (CPB) (without arrest). Complete excision was achieved without myocardial loss or left ventricular communication. Hepatic cysts were removed 1 month later. Albendazole was continued for 3 months postoperatively with weekly liver function test (LFT) monitoring. Full recovery was observed at 6 months, and a 24-month surveillance plan was established.

**Clinical discussion:**

Cardiac hydatid cysts are often asymptomatic until rupture. TTE is first-line; CT (or MRI) aids in surgical planning. In multi-organ disease, cardiac resection is prioritized. Intraoperative hypotension may mandate CPB, even without pre-existing instability. Perioperative albendazole reduces recurrence.

**Conclusion:**

Synchronous cardiac and hepatic hydatidosis in children requires a multidisciplinary staged approach prioritizing cardiac resection. Complete excision without myocardial loss, CPB if hemodynamic compromise occurs during exposure, and perioperative albendazole (15 mg/kg/day with LFT monitoring) are safe and effective, ensuring full recovery and preventing recurrence.

## Introduction

Hydatidosis is a parasitic disease caused by the tapeworm Echinococcus granulosus. The disease is prevalent in various regions around the world, including the temperate climates of Asia, the Mediterranean, Australia, and South America^[^1^]^. It primarily affects animals, especially livestock and pets. Humans may become secondary hosts through close interaction with infected animals or by consuming food and water that have been contaminated with the parasite^[^2^]^. The liver is the most targeted organ by this parasite (70%), followed by the lungs (20%). Other organs that may also be infected include the heart (0.5–2%), kidneys, spleen, and muscles^[^3^]^. While clinical symptoms may vary depending on the location of the cyst, they are usually non-specific ^[^4^]^. The placement of the cyst in the heart is usually asymptomatic, but it may cause dyspnea, chest pain, and palpitations. On the other hand, serious complications might occur if the cardiac cyst ruptures, including pericardial tamponade, embolism, and an anaphylactic reaction^[^5^]^. Transthoracic echocardiography (TTE) remains the first diagnostic tool, while computed tomography (CT) and magnetic resonance imaging (MRI) can provide more anatomic details necessary for a surgical plan. Herein, an 8-year-old male was diagnosed with multiple hydatid cysts in his liver and a pericardial cyst, with the pericardial cyst compressing his left ventricle.

HIGHLIGHTS

- Maintain a high index of suspicion for synchronous cardiac and hepatic hydatidosis in pediatric patients from endemic regions, as cardiac involvement – though rare – carries a significant risk of life-threatening complications.
- Adopt a staged surgical approach that prioritizes the resection of the cardiac cyst, supported by perioperative albendazole therapy, to ensure patient safety and prevent recurrence.

This case report has been presented in line with the SCARE checklist^[^6^]^

## Case presentation

**Figure 1.**
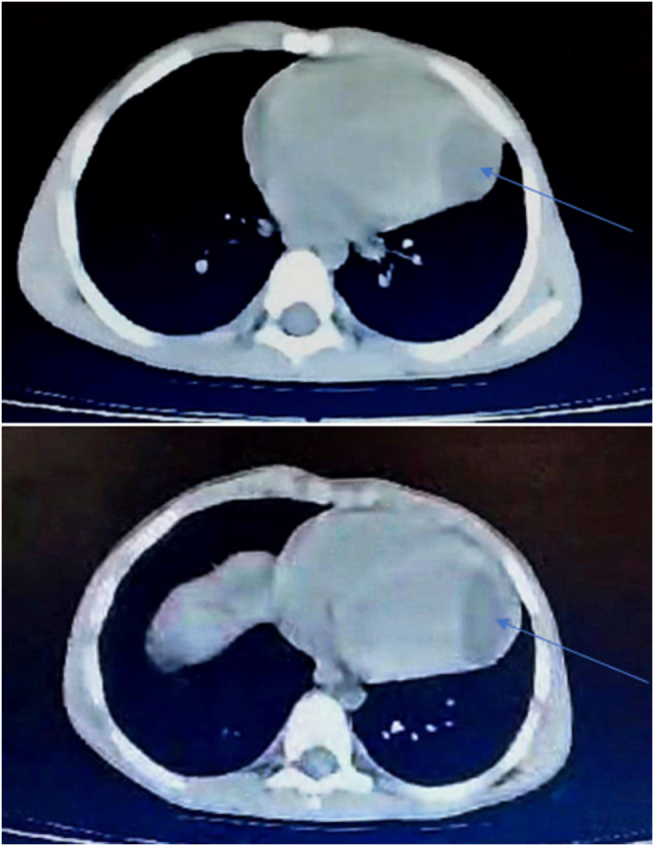
CT image showing the cardiac hydatid cyst and hepatic hydatid cyst (arrow).

**Figure 2.**
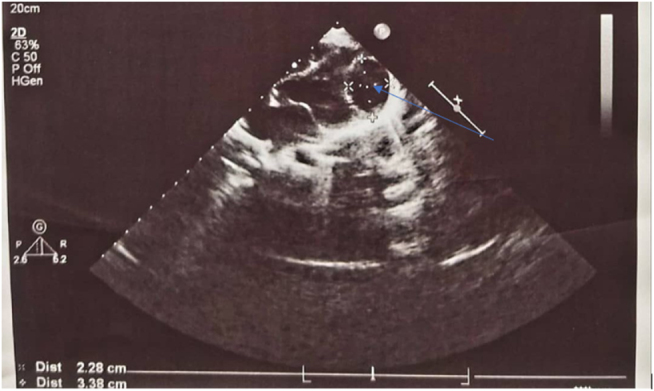
Transthoracic echocardiogram image showing the cardiac cyst (arrow).

An 8-year-old Arab male was admitted to our hospital with abdominal pain, recurrent vomiting, fever, and a dry cough. There was no history of hemodynamic instability or arrhythmia related to the cardiac cyst. Physical examination revealed hepatomegaly approximately 1 cm below the right costal margin. A chest X-ray showed an abnormal cardiac silhouette with a calcified lesion. Abdominal ultrasound showed about seven cystic masses in the liver. Contrast-enhanced CT of the chest, abdomen, and pelvis was performed; however, cardiac MRI was not available at our institution. CT detected a low-density, oval-shaped lesion with calcified walls at the cardiac apex (Fig. 1), and the liver was found to be enlarged with some calcified cysts in its stroma. The cardiac cyst was localized to the subepicardial region of the left ventricle, with no involvement of the coronary arteries. TTE revealed a cyst in the pericardium compressing the left ventricle (Fig. 2).

**Figure 3.**
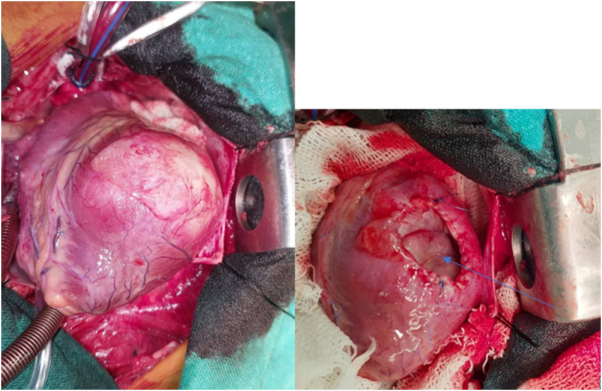
Intraoperative image showing the cardiac hydatid cyst after raising the heart and the cavity after the removal of the cardiac hydatid cyst (arrow).

Albendazole was started at a dose of 15 mg/kg/day (divided into two daily doses) for 2 weeks prior to the first surgery. The decision was made to resect the cardiac cyst as the first intervention. The operation was performed via a median sternotomy. The pericardium was opened, and the cyst was apparent on the left ventricle (Fig. 3). However, during initial manipulation to expose the cyst, the patient developed hypotension, precluding safe mobilization without circulatory support. Therefore, total cardiopulmonary bypass (CPB) was initiated to maintain hemodynamic stability and allow complete decompression of the heart for optimal exposure. The heart was not arrested; it continued beating on CPB. The cyst fluid was aspirated with a syringe to reduce tension, and a scolicidal agent (hypertonic saline 20%) was injected into the cyst and left in contact for 5 minutes before enucleation. The cyst was completely excised (total cystectomy), and no myocardial layer was lost during the procedure. There was no communication between the cyst cavity and the left ventricular cavity. The margins of the cavity created by cyst removal were sutured with continuous sutures for hemostasis (Fig. 3). The patient was weaned off CPB uneventfully. The patient convalesced well in the intensive care unit and was discharged on albendazole (15 mg/kg/day) to be continued for three months.

One month after the cardiac surgery, the patient remained clinically stable with no interim complications. Liver function tests were monitored weekly and remained within normal limits. He then underwent surgical removal of the hepatic cysts via a standard laparotomy approach. At a 6-month follow-up, he remained asymptomatic and showed no evidence of recurrence of the hydatid cysts.

## Discussion

Hydatidosis is an animal-transmitted infection caused by the cestode *E. granulosus*, and human beings are only intermediate hosts. The World Health Organization reports that rural areas in endemic regions contain the highest rates of human cystic echinococcosis; these rates might reach up to 5–10%^[^7^]^. This disease is characterized by the development of cysts, known as hydatid cysts, that are formed inside the organs of intermediate hosts, such as humans. It is transmitted from primary hosts after they excrete the cestode eggs through their feces, and then humans accidentally ingest the eggs through contaminated food and water. Those primary hosts include dogs, cats, and livestock^[^8^]^. In our case, the patient lived in a rural area with close contact with livestock, but there was no reported history of consuming uncooked meat. Only 0.5–2% of cases have cardiac involvement, which may affect various heart structures, with the primary location being the left ventricle (up to 34% of cases), followed by the interventricular septum (20.8%), the right ventricle (16.2%), the pericardium (11.7%), the right atrium (7.3%), and the left atrium (4.9%)^[^9^]^. In this patient, the cyst was located in the subepicardial region of the left ventricle with no involvement of the coronary arteries, a distinction that is critical for surgical planning.

Several imaging modalities can be utilized to diagnose cardiac hydatid cysts; however, TTE is often preferred as a first-line imaging technique because it is widely available and has excellent sensitivity. CT and MRI are particularly valuable in assessing the anatomic relation of the cyst with the surrounding tissues and thus provide important information for the surgeon^[^9–12^]^. Cardiac MRI was not available at our institution; nevertheless, contrast-enhanced CT sufficiently delineated the cyst’s subepicardial location and its relation to the left ventricle and coronary arteries, guiding the surgical approach. Cardiac hydatid cysts are usually silent until they begin to enlarge. As they grow, patients may experience various symptoms, including chest pain (the most frequently reported symptom), dyspnea, palpitations, and syncope. Moreover, patients might face many life-threatening complications, such as heart failure, anaphylactic reactions, arrhythmias, and pericardial tamponade^[^10,11^]^. In the present case, the patient had no prior history of hemodynamic instability or arrhythmia attributable to the cardiac cyst, highlighting that even asymptomatic or minimally symptomatic cysts warrant timely intervention.

Surgical removal of the cardiac hydatid cyst should not be delayed, even in asymptomatic patients, since cyst rupture may lead to fatal complications such as anaphylactic shock and pericardial tamponade. Anti-hydatid medications (albendazole or mebendazole) play a supplementary role in surgery to decrease the possibility of recurrence^[^13–16^]^. In our patient, albendazole was administered at 15 mg/kg/day (divided into two daily doses) for 2 weeks preoperatively and continued for 3 months postoperatively, with weekly monitoring of liver enzymes, which remained within normal limits. The decision to perform the cardiac cystectomy first was based on the higher risk of fatal complications (rupture, tamponade, embolism) from the cardiac lesion.

The surgical technique merits detailed discussion. The cyst was completely excised (total cystectomy) without loss of any myocardial layer, and there was no communication between the cyst cavity and the left ventricular cavity. A scolicidal agent (hypertonic saline 20%) was injected into the cyst and left in contact for 5 minutes before enucleation to reduce the risk of spillage and secondary seeding. While injecting scolicidal agents into a cardiac cyst carries a theoretical risk of thrombosis or embolic shower if the agent enters the systemic circulation, this risk is minimized by first aspirating cyst fluid to decrease tension, ensuring the injection needle remains within the cyst cavity, and using a short contact time. No such complication occurred in this case. Although the cyst wall was partially calcified, this does not necessarily indicate a non-viable cyst; viable protoscolices may still be present, and therefore surgical removal remains indicated.

The use of CPB was not planned preemptively but became necessary intraoperatively. During initial attempts to expose the cyst by manipulating the heart, the patient developed significant hypotension, precluding safe continuation without circulatory support. CPB was therefore instituted to maintain hemodynamic stability, allowing complete decompression of the heart and optimal exposure for safe cyst enucleation. The heart was not arrested but continued beating on CPB, which preserved myocardial function and avoided ischemic injury. This approach is particularly valuable in pediatric patients, whose smaller cardiac chambers and greater hemodynamic lability make manipulation without support more hazardous.

One month after cardiac surgery, the patient underwent hepatic cyst resection without complications. The staged approach minimized physiological insult. At 6 months, the patient was asymptomatic with no recurrence. Per guidelines, follow-up extends to 24 months with serial imaging. Pediatric considerations include long life expectancy, aggressive treatment, vigilant surveillance, weight-based albendazole dosing, and hepatotoxicity monitoring. This case shows that staged surgery (cardiac cyst resection with CPB if needed, then hepatic removal), plus perioperative albendazole, is safe and effective for children with synchronous cardiac and hepatic hydatidosis.

## Conclusion

Combined cardiac and hepatic hydatidosis is rare, especially in children. In endemic regions, a high index of suspicion for multi-organ involvement is essential. Successful management relied on multimodal imaging (TTE and CT; cardiac MRI not required) and a staged surgical strategy prioritizing cardiac cyst resection to prevent rupture or embolism. Despite no preoperative instability, intraoperative manipulation of a subepicardial cyst may cause hypotension, necessitating CPB (without arrest) for safe excision. The cyst was fully removed without myocardial loss or left ventricular communication. Hypertonic saline (20%, 5 min) was used as a scolicidal agent with no thrombosis. Partially calcified cysts should still be resected due to possible viability. Perioperative albendazole (15 mg/kg/day for 2 weeks pre- and 3 months postoperatively, with weekly liver function monitoring) was safe and effective, yielding complete recovery and no recurrence at 6 months. Extended follow-up with serial imaging up to 24 months is planned, particularly important in children given their long life expectancy.

## Data Availability

The data that support the findings of this study are available from the corresponding author upon reasonable request.
